# Healthcare costs attributable to abnormal weight in China: evidence based on a longitudinal study

**DOI:** 10.1186/s12889-023-16855-6

**Published:** 2023-10-05

**Authors:** Shiqi Zhao, Xinpeng Xu, Hua You, Jinjin Ge, Qifeng Wu

**Affiliations:** 1 Department of Social Medicine and Health Education, School of Public Health, Nanjing Medical University, 101 Longmian Avenue, Nanjing, 211166 Jiangsu P.R. China; 2 Institute of Healthy Jiangsu Development, Nanjing Medical University, Nanjing, China

**Keywords:** Overweight, Obesity, Underweight, Healthcare costs, China

## Abstract

**Background:**

The prevalence of abnormal weight is on the rise, presenting serious health risks and socioeconomic problems. Nonetheless, there is a lack of studies on the medical cost savings that can be attained through the mitigation of abnormal weight. The aim of this study was to estimate the impact of abnormal weight on healthcare costs in China.

**Methods:**

The study employed a 4-wave panel data from China Family Panel Studies (CFPS) between 2012 and 2018 (11,209 participants in each wave). Inpatient, non-inpatient and total healthcare costs were outcome variables. Abnormal weight is categorized based on body mass index (BMI). Initially, the two-part model was employed to investigate the impact of overweight/obesity and underweight on healthcare utilisation and costs, respectively. Subsequently, the estimated results were utilised to calculate the overweight/obesity attributable fraction (OAF) and the underweight attributable fraction (UAF).

**Results:**

In 2018, healthcare costs per person for overweight and obese population were estimated to be $607.51 and $639.28, respectively, and the underweight population was $755.55. In comparison to people of normal weight, individuals who were overweight/obese (OR = 1.067, p < 0.05) was more likely to utilise healthcare services. Overweight/obesity attributable fraction (OAF) was 3.90% of total healthcare costs and 4.31% of non-inpatient costs. Overweight/obesity does not result in additional healthcare expenditures for young people but increases healthcare costs for middle-aged adults (OAF = 7.28%) and older adults (OAF = 6.48%). The non-inpatient cost of underweight population was significantly higher than that of normal weight population (β = 0.060,p < 0.1), but the non-inpatient health service utilisation was not significantly affected.

**Conclusions:**

Abnormal weight imposes a huge economic burden on individuals, households and the society. Abnormal weight in Chinese adults significantly increased healthcare utilisation and costs, particular in non-inpatient care. It is recommended that government and relevant social agencies provide a better social environment to enhance individual self-perception and promote healthy weight.

## Background

The issue of abnormal body weight poses a major concern for population health. Presently, there are current more than 2 billion individuals globally who are either overweight or obese [1, 2], while underweight is prevalent among the elderly and women [3–5]. According to a study published in 2021, China has the largest population of overweight individuals (34.3%) and obese individuals (16.4%) worldwide, totaling over 600 million people [6]. Notably, the majority of underweight adults are concentrated in the under-30 and over-60 age groups, particularly among women and older adults residing in rural areas [7]. This holds significance as variations in body mass index (BMI) have been found to impact the mortality risk in adults, with both higher and lower BMI values being associated with an increased mortality risk. Specifically, the risk of mortality linked to being underweight is attributed to neurological diseases and accidents [8].

The financial implications stemming from abnormal weight extend beyond individual patients [9, 10], encompassing the healthcare system as well, despite the fact that certain expenses are covered by health insurance [11, 12]. Abnormal weight not only constitutes an independent disease, resulting in healthcare costs [13], but it also serves as a major risk factor for a number of chronic conditions, such as hypertension, type 2 diabetes, cerebrovascular disease, and cancer [14], thereby contributing to substantial healthcare expenditures [15, 16]. Examining the policy regarding the management of abnormal weight requires a comprehensive understanding of the healthcare costs associated with being overweight/obese or underweight, as well as the proportion of these costs attributable to this condition. These figures aid in converting the negative health effects of abnormal weight into measurable costs and ratios that more precisely depict their impact [17, 18].

Prior research conducted by Chinese academics has endeavored to increase awareness among healthcare providers regarding overweight and obesity by estimating the substantial economic burden of prevalent chronic diseases resulting from excessive weight [19, 20]. However, there are several issues about the analysis. Firstly, the effects of abnormal weight on physical health are not well-defined and vary significantly among different groups of population. In addition, considering only the healthcare costs associated with a limited number of major chronic diseases may not provide a comprehensive evaluation of the overall financial burden caused by abnormal body weight. Using mixed cross-sectional data from the years 2000 to 2009, Qin et al. (2016) estimated that overweight/obesity contributed 24.35 billion CNY to the healthcare costs of Chinese adults [21]. Nevertheless, it is important to note that the data used in that study are relatively out of date, and given the escalating prevalence of overweight/obesity, the findings may not accurately reflect the current situation [22]. For instance, a 2018 study of Japanese adults between the ages of 40 and 69 found that 9.62% of healthcare costs in this group were attributable to overweight and obesity, which is a significant increase from 3.20% found in a 2002 study [23]. In addition, previous studies have paid less attention to underweight populations, and there is a lack of reports concerning underweight populations in China.

This study investigates the impact of overweight/obesity and underweight on healthcare expenditures, specifically focusing on inpatient costs, non-inpatient costs, and total healthcare costs. The study utilises data from the China Family Panel Study (CFPS), a nationally representative longitudinal dataset, to provide recent insights. Notably, this study contributes to the existing literature by providing updated estimates of medical costs attributable to overweight/obesity as well as addressing the dearth of information regarding the prevalence and associated costs of underweight among Chinese adults. In addition, we utilised econometric modeling, a reliable technique extensively employed in studies estimating healthcare costs. The results of this study will serve as a foundation for designing and implementing more effective weight control measures.

## Methods

### Data source

This study utilised the panel data provided by the latest four waves of data (2012, 2014, 2016, 2018) of China Family Panel Studies (CFPS). CFPS is a national comprehensive family social tracking survey conducted by the Institute of Social Science Survey (ISSS) of Peking University. The survey was conducted using a multi-stage, equal probability sampling technique, covering 25 provinces and 94.5% of the adult population in China, collecting information on education, health behaviors and economic activities. The Peking University Biomedical Ethics Review Committee provided ethical approval of the survey (IRB00001052-14010). All respondents read a statement describing the purpose of the study and provided their agreement to continue. More details about the CFPS are available from studies [24–27].

In 2010, The CFPS initiated a baseline survey and has subsequently followed individuals every two years. The individual-level data sets currently available to the publicly include the years 2010, 2012, 2014, 2016, 2018 and 2020. In this study, we mainly used information on healthcare utilisation, healthcare costs, health behaviors, sociodemographic characteristics, as well as socioeconomic status for the analysis. The 2010 survey on healthcare information only included the utilisation and costs of inpatient services, the 2012–2020 survey was expanded to include non-inpatient services. In addition, the COVID-19 pandemic had a significant impact on the likelihood and cost of utilising healthcare services in 2020. To rule out unreasonable changes in healthcare utilisation and expenditures caused by the COVID-19 pandemic, we utilised data from 2012, 2014, 2016, and 2018. Following the removal of missing values for key variables, the final sample used for analysis consisted of 44,836 observations. (11,209 individuals each year).

### Measurement

#### Healthcare utilisation and costs

The main dependent variables in this study were annual healthcare utilisation (individuals with positive healthcare expenses) and total healthcare costs. Annual total healthcare cost was calculated by adding the inpatient costs and non-inpatient costs. Inpatient costs include expenses for laboratory tests, consultations, medicines, bed tariffs, and nursing care. The non-inpatient costs refer to the expenses incurred as a result of the patient’s illness other than the inpatient costs. For healthcare costs, first, costs were inflation-adjusted to the year 2018 Chinese Yuan (CNY) by using national Consumer Price Index (CPI) of each corresponding year [28]. Then, to facility cross-country comparisons, the costs were converted into USD of the year 2018.The currency exchange rate between US dollars and Chinese Yuan was: 1.0 USD = 6.6174 CNY in 2018.

#### Abnormal weight

The key independent variables in this study were overweight/obesity and underweight based on BMI category. BMI is defined as the weight in kilograms divided by the height in meters squared. The criteria for the BMI were applicate according to the National Health and Family Planning Commission of the People’s Republic of China [29]. Specifically, it is divided into four categories: underweight (< 18.5 kg/m^2^), normal weight (18.5–23.9 kg /m^2^), overweight (24.0-27.9 kg /m^2^) and obesity (≥ 28.0 kg /m^2^). In this study, we focused on the healthcare costs associated with abnormal weight as compared with normal weight. We combined obesity and overweight into a category termed overweight/obesity.

#### Covariates

In the model, we accounted for the factors that may affect the utilisation and cost of healthcare services for individuals based on previous studies [23, 30, 31]. Included were demographic characteristics (age, gender, location, marital status and educational level), health behaviors (smoking status, drinking status, health status), socioeconomic status (health insurance and income level).

Geographical locations are classified into two distinct categories, namely urban and rural, based on the criteria for the division by the National Bureau of Statistics; Marital status is divided into two categories: cohabited (married or cohabitating) and other (single or separated or divorced or widowed); Educational level is divided into three categories: primary school and below, junior high school, and senior high school and above; Smoking status, drinking status were all dichotomous variables (0 = No, 1 = Yes); Health status is the self-rated health status of the respondent (1 = good, 2 = general, 3 = bad); health insurance is a multicategorical variable (0 = None, 1 = Publicly-funded medical care, 2 = Urban employee basic medical insurance, 3 = Urban resident basic medical insurance, 4 = Supplementary medical insurance, 5 = New rural cooperative medical scheme); Considering the consistency of indicators in CFPS each wave, the income level adopted in this study is primarily based on self-reported subjective income level, which is evaluated by individuals based on their local location of household income, ranging from very low “1” to very high “5”.

### Statistical analysis

First, the mean (standard deviation) and frequency (percentage) were used to conduct a descriptive statistical analysis of each variable year by year. Second, in order to explore the impact of abnormal weight on healthcare utilisation and costs among adults, and to predict the healthcare costs attributable to abnormal weight, we developed the following two-part model based on existing studies [32], which is a standard health economics method for estimating healthcare demand [33].

Notably, for healthcare costs for which there is a large amount of zero-valued data, the two-part model does not rely on assumptions of homoskedasticity and normality of the outcome variable [25, 33]. Using as an example the estimated equation for overweight/obesity:

1 $$\text{D}\text{E}{\text{x}\text{p}}_{it}={\gamma }_{0}+{\gamma }_{1}{\text{O}}_{it}+{\gamma }_{2}{X}_{it}+{\alpha }_{i}+{u}_{it}$$

2 $$\text{I}\text{n}{Exp}_{it}={\beta }_{0}+{\beta }_{1}{\text{O}}_{it}+{\beta }_{2}{X}_{it}+{{\alpha }^{{\prime }}}_{i}+{{u}^{{\prime }}}_{it}$$

A logit model was established for $\text{D}\text{E}{\text{x}\text{p}}_{it}$ (Eq. (1)), denoting $\text{D}\text{E}{\text{x}\text{p}}_{it}$ as the healthcare services utilisation of the i^th^ indivudual in year t: when the individual utilised healthcare services (that is, positive healthcare costs occurred), the value of $\text{D}\text{E}{\text{x}\text{p}}_{it}$ was 1, and 0 otherwise. ${\text{O}}_{it}$ is the primary independent variable Overweight/Obese, ${X}_{it}$ represents the covariate matrix made up of individual demographic characteristics, health behaviors and socioeconomic status, ${\alpha }_{i}$ is the random heterogeneity of the i^th^ observation that does not vary over time, and ${\text{u}}_{\text{i}\text{t}}$ is the random error term.

And then, a linear regression model (Eq. (2)) is established for $\text{I}\text{n}{Exp}_{it}$. Under the condition of $\text{D}\text{E}{\text{x}\text{p}}_{it}$=1, that is, among the population with healthcare utilisation, denoted $\text{I}\text{n}{Exp}_{it}$ as the natural logarithm of the healthcare costs of the i^th^ individual in year t, the remaining variables are specified identically to those in Eq. 1.

The parameters estimated by the two preceding equations allowed us to predict the healthcare costs attribute to overweight/obesity. First, the healthcare costs of each observation in the actual situation can be predicted based on the estimated coefficient of each variable and the actual characteristics of the individual, which is called the actual predicted value (${\widehat{\text{Y}}}_{\text{i}}^{\text{A}}$); Second, it was assumed that the body weight of overweight/obesity individuals in the sample was within the normal range (i.e. overweight/obesity = 0), and other characteristics remained unchanged. The estimated parameters of the model can be used to obtain the healthcare costs of each individual under normal weight, which is the counterfactual value of healthcare costs ${\widehat{\text{Y}}}_{\text{i}}^{\text{C}\text{F}}$; Finally, the overweight/obesity attributable fraction (OAF) in healthcare costs can be calculated by Eq. (3) using the predicted actual and counterfactual values.

3 $$OAF=\frac{{\widehat{Y}}_{i}^{A}-{\widehat{Y}}_{i}^{CF}}{{\widehat{Y}}_{i}^{A}}$$

Similarly, the effect of underweight on healthcare costs can be explored and the underweight attributable fraction (UAF) calculated by assigning the primary independent variable to underweight (${\text{U}}_{it}$). Notably, healthcare costs attributable to overweight/obesity and underweight were estimated separately. In order to estimating healthcare costs associated with overweight/obesity, we excluded underweight samples and used normal weight as the reference group. Similarly, when evaluating healthcare costs attributable to underweight, we excluded the overweight/obese sample and used the normal weight as the reference group. The heterogeneity of different age groups was then analyzed. We divided age into three groups (18–44, 45–59, and 60 and above) to estimate the parameters of healthcare service utilisation and cost of abnormal weight and the OAF value, respectively. All data were analyzed using Stata software 16.0 (Stata Corp., College Station, TX, USA).

## Results

### Characteristics of participants

Descriptive statistics of variables are presented in Table 1. At the baseline, the mean (SD) age of the sample was 47.66(12.92) years; 51.54% (5,777/11,209) of the participants are male; 28.20% of the participants are urban residents; 50.88% of the participants achieved primary school education or below; 91.61% of the participants live with their spouses, and 90.08% of them were covered by health insurance. In terms of BMI, 27.56% and 7.27% of adults were overweight and obese respectively in 2012, both of which exhibited an upward tendency, with the proportions rising to 33.05% and 9.80% in 2018. In contrast, the prevalence of underweight decreased from 7.20% in 2012 to 5.33% in 2018. In terms of the healthcare utilisation, the inpatient services utilisation rate was 8.36% in 2012, indicating a general upward trend, and it was 15.21% in 2018. From 2012 to 2018, the non-inpatient healthcare utilisation rate fell from 77.26 to 67.65%, and total healthcare utilisation rate decreased from 79.45 to 71.07%.

**Table 1:** Descriptive statistics of study sample [n (%)] (China. 2012–2018)

Type of variable	2012	2014	2016	2018
BMI				
Underweight	807 (7.20)	686 (6.12)	731 (6.52)	597 (5.33)
Normal weight	6498 (57.97)	6230 (55.58)	6064 (54.10)	5809 (51.82)
Overweight	3089 (27.56)	3359 (29.97)	3376 (30.12)	3705 (33.05)
Obesity	815 (7.27)	934 (8.33)	1038 (9.26)	1098 (9.80)
Age, Mean (SD)	47.66 (12.92)	49.66 (12.92)	51.66 (12.92)	53.66 (12.93)
Male	5777 (51.54)	5778 (51.55)	5778 (51.55)	5775 (51.52)
Urban	3161 (28.20)	3237 (28.88)	3276 (29.23)	3269 (29.16)
Education level				
Prime and below	5703 (50.88)	5703 (50.88)	5703 (50.88)	5703 (50.88)
Middle	3291 (29.36)	3291 (29.36)	3272 (29.19)	3167 (28.25)
High and above	2215 (19.76)	2215 (19.76)	2234 (19.93)	2339 (20.87)
Married	10,269 (91.61)	10,259 (91.52)	10,251 (91.45)	10,153 (90.58)
Smoke	3673 (32.77)	3619 (32.29)	3443 (30.72)	3526 (31.46)
Drink	2037 (18.17)	2048 (18.27)	2014 (17.97)	2051 (18.30)
Insurance	10,097 (90.08)	10,524 (93.89)	10,550 (94.12)	10,572 (94.32)
None	1112 (9.92)	685 (6.11)	659 (5.88)	637 (5.68)
Publicly-funded medical care	441 (3.93)	356 (3.18)	252 (2.25)	280 (2.50)
Urban employee basic medical insurance	1404 (12.53)	1655 (14.76)	1679 (14.98)	1745 (15.57)
Urban resident basic medical insurance	694 (6.19)	886 (7.90)	930 (8.30)	1025 (9.14)
Supplementary medical insurance	34 (0.30)	61 (0.54)	36 (0.32)	56 (0.50)
New rural cooperative medical scheme	7524 (67.12)	7566 (67.50)	7653 (68.28)	7466 (66.61)
Health status				
Good	2991 (26.68)	3495 (31.18)	2946 (26.28)	2712 (24.19)
General	4014 (35.81)	4213 (37.59)	4017 (35.84)	4577 (40.83)
Bad	4204 (37.51)	3501 (31.23)	4246 (37.88)	3920 (34.97)
Income level				
Income level 1	2872 (25.62)	1887 (16.83)	2242 (20.00)	1093 (9.75)
Income level 2	3528 (31.47)	2663 (23.76)	3043 (27.15)	1983 (17.69)
Income level 3	4064 (36.26)	5401 (48.18)	4866 (43.41)	5393 (48.11)
Income level 4	510 (4.55)	881 (7.86)	692 (6.17)	1575 (14.05)
Income level 5	235 (2.10)	377 (3.36)	366 (3.27)	1165 (10.39)
Healthcare utilisation				
Inpatient	937 (8.36)	1212 (10.81)	1399 (12.48)	1705 (15.21)
Non-Inpatient	8660 (77.26)	7376 (65.80)	7589 (67.70)	7583 (67.65)
Healthcare	8906 (79.45)	7719 (68.86)	7893 (70.42)	7966 (71.07)
Healthcare cost ($),Mean (SD)				
Inpatient	146.05 (1042.85)	214.60 (1264.17)	255.91 (1584.17)	348.66 (1742.59)
Non-Inpatient	172.53 (535.55)	190.49 (645.91)	209.37 (634.57)	247.03 (1019.63)
Healthcare	318.58 (1233.71)	405.09 (1513.74)	465.28 (1791.30)	595.69 (2184.16)
Sample size	11,209	11,209	11,209	11,209

Note: All expenditures are based on 2018 CNY, adjusted for CPI from 2012 to 2018, and converted to USD based on an average exchange rate of 6.6174 in 2018

From 2012 to 2018, both inpatient and non-inpatient costs exhibited an upward trend (Table 1). The same pattern was observed across different BMI categories. Particularly, the inpatient costs of underweight individuals were higher than those of normal weight and overweight/obese individuals (Table 2).

**Table 2:** The average healthcare cost of different types of health services among different BMI groups ($)

Year	Inpatient cost	Non-Inpatient cost	Total healthcare cost
**Normal weight**			
2012 (N = 6498)	142.76 (1063.43)	154.79 (414.78)	297.55 (1193.05)
2014 (N = 6230)	189.60 (1080.12)	176.74 (573.28)	366.34 (1327.45)
2016 (N = 6064)	245.57 (1608.28)	197.63 (578.59)	443.20 (1804.33)
2018 (N = 5809)	327.22 (1674.00)	236.27 (1208.46)	563.49 (2235.46)
**Underweight**			
2012 (N = 807)	170.81 (1078.69)	179.85 (719.26)	350.66 (1358.35)
2014 (N = 686)	304.87 (2198.32)	190.21 (433.93)	495.09 (2252.46)
2016 (N = 731)	341.53 (2062.89)	251.91 (703.46)	593.45 (2194.72)
2018 (N = 597)	482.01 (2085.07)	273.54 (672.17)	755.55 (2248.78)
**Overweight**			
2012 (N = 3089)	138.01 (986.40)	197.98 (682.81)	335.98 (1277.95)
2014 (N = 3359)	235.61 (1314.43)	203.70 (808.74)	439.31 (1644.14)
2016 (N = 3376)	274.66 (1533.64)	214.48 (725.77)	489.14 (1788.32)
2018 (N = 3705)	354.90 (1702.34)	252.61 (764.23)	607.51 (2071.03)
**Obesity**			
2012 (N = 815)	178.29 (1050.16)	210.23 (537.25)	388.51 (1250.29)
2014 (N = 934)	239.47 (1276.76)	234.93 (567.00)	474.40 (1510.88)
2016 (N = 1038)	195.03 (1157.60)	231.35 (574.11)	426.38 (1352.43)
2018 (N = 1098)	368.54 (2007.26)	270.74 (819.09)	639.28 (2243.02)
**Overweight/obesity**			
2012 (N = 3904)	146.41 (1000.04)	200.53 (655.06)	346.95 (1272.24)
2014 (N = 4293)	236.45 (1306.18)	210.49 (762.74)	446.94 (1615.98)
2016 (N = 4414)	255.94 (1454.25)	218.45 (693.07)	474.38 (1695.99)
2018 (N = 4803)	358.02 (1776.45)	256.75 (777.06)	614.77 (2111.39)

Note: All expenditures are based on 2018 CNY, adjusted for CPI from 2012 to 2018, and converted to USD based on an average exchange rate of 6.6174 in 2018

### The impact of overweight/obesity on healthcare utilisation and healthcare costs

Using a two-part model as previously described, we analyzed the impact of overweight/obesity on healthcare utilisation and costs (Table 3). Overweight/obesity individuals were more likely to utilise healthcare services (OR = 1.067, p < 0.05) and non-inpatient services (OR = 1.070, p < 0.05). In terms of healthcare costs, those with overweight/obesity have higher healthcare costs (β = 0.082, p < 0.01) and non-inpatient costs (β = 0.088, p < 0.01) than people of normal weight. However, the effect of overweight/obesity on the increase in inpatient costs was not statistically significant (p > 0.1).

**Table 3:** Parametric estimates of healthcare utilisation and expenditure due to overweight/obesity (China. 2012–2018)

Type of characteristic		Annual healthcare utilisation [OR]			Annual healthcare costs^a^ [β]		
		Non-inpatient	Inpatient	Total healthcare	Non-inpatient	Inpatient	Total healthcare
Weight status (Ref: Normal)	Overweight/obesity	1.070**	1.036	1.067**	0.088***	0.022	0.082***
Age, years	Age	1.023***	1.034***	1.027***	0.018***	0.002	0.021***
Gender (Ref: Female)	Male	0.654***	1.045	0.672***	-0.114***	0.331***	-0.015
Educational level (Ref: Primary and below)	Middle	0.875***	0.973	0.883***	-0.043	-0.001	-0.034
	High and above	0.969	0.971	0.969	-0.080	0.013	-0.077**
Marital status (Ref: Other)	Cohabited	0.912	0.914	0.876**	0.018	0.141**	0.023
Location (Ref: Rural)	Urban	0.935	1.060	0.956	0.147***	0.206***	0.161***
Drinking status (Ref: no)	Yes	0.873***	0.728***	0.825***	-0.165***	-0.294***	-0.245***
Smoking status (Ref: no)	Yes	0.972***	0.750***	0.908**	-0.138***	-0.261***	-0.237***
Health insurance (Ref: none)	Publicly-funded medical care	1.059	1.964***	1.102	0.267***	0.264**	0.370***
	Urban employee basic medical insurance	1.331***	1.754***	1.406***	0.184***	0.130	0.256***
	Urban resident basic medical insurance	1.082	1.532***	1.186**	0.058	0.130	0.159***
	Supplementary medical insurance	1.139	1.187	1.149	-0.054	-0.018	-0.101
	New rural cooperative medical scheme	1.229***	1.393***	1.309***	-0.049	0.012	0.004
Health status (Ref: Good)	General	2.066***	1.734***	2.116***	0.310***	0.027	0.328***
	Bad	3.934***	4.395***	4.820***	0.868***	0.250	1.020***
Income level (Ref: Income level 1)	Income level 2	1.142***	0.881**	1.112**	-0.084***	-0.201***	-0.132***
	Income level 3	1.100**	0.839***	1.051	-0.117***	-0.120***	-0.166***
	Income level 4	1.206***	0.924	1.151**	-0.123***	-0.089	-0.149***
	Income level 5	0.949	0.993	0.904	0.057	-0.085	0.009
Province	Province	Yes	Yes	Yes	Yes	Yes	Yes
Year	Year	Yes	Yes	Yes	Yes	Yes	Yes

Note: * p < 0.1, ** p < 0.05, *** p < 0.01

^a^ Annual healthcare costs are the natural logarithm of actual costs

### The impact of underweight on healthcare utilisation and healthcare costs

Similarly, we utilised a two-part model to analyze the impact of underweight on healthcare utilisation and costs (Table 4). There was no significant difference in the probability of healthcare utilisation between the underweight population and the normal weight population. In the cost estimation, the underweight population had significantly higher non-inpatient costs (β = 0.060, p < 0.1) than the normal weight group, but there was no statistically significant difference in inpatient costs and healthcare costs (p > 0.1).

**Table 4:** Parametric estimates of healthcare utilisation and expenditure due to underweight (China. 2012–2018)

Type of characteristic		Annual healthcare utilisation [OR]			Annual healthcare costs^a^ [β]		
		Non-inpatient	Inpatient	Total healthcare	Non-inpatient	Inpatient	Total healthcare
Weight status (Ref: Normal)	Underweight	1.048	1.003	1.033	0.060*	0.024	0.058
Age, years	Age	1.018***	1.031***	1.022***	0.013***	0.001	0.017***
Gender (Ref: Female)	Male	0.657***	1.045	0.694***	-0.108***	0.322***	0.001
Educational level (Ref: Primary and below)	Middle	0.918*	0.960	0.920	-0.042	-0.028	-0.038
	High and above	1.019	0.912	1.003	-0.124***	0.001	-0.128***
Marital status (Ref: Other)	Cohabited	0.874**	0.930	0.851**	-0.006	0.143**	0.009
Location (Ref: Rural)	Urban	0.962	0.983	0.984	0.122***	0.217***	0.124***
Drinking status (Ref: no)	Yes	0.883**	0.703***	0.822***	-0.129***	-0.362***	-0.225***
Smoking status (Ref: no)	Yes	0.919*	0.801***	0.846***	-0.142***	-0.255***	-0.233***
Health insurance (Ref: none)	Publicly-funded medical care	0.979	2.312***	1.053	0.311***	0.222	0.454***
	Urban employee basic medical insurance	1.214**	1.763***	1.282***	0.191***	0.239**	0.271***
	Urban resident basic medical insurance	0.985	1.764***	1.111	0.055	0.214(0.1)	0.170***
	Supplementary medical insurance	1.202	1.210	1.189	-0.084	-0.056	-0.106
	New rural cooperative medical scheme	1.236***	1.306**	1.288***	-0.058	0.034	-0.014
Health status (Ref: Good)	General	2.038***	1.642***	2.065***	0.312***	-0.004	0.324***
	Bad	4.003***	4.067***	4.958***	0.886***	0.280***	1.026***
Income level (Ref: Income level 1)	Income level 2	1.153***	0.826***	1.097*	-0.097***	-0.121**	-0.143***
	Income level 3	1.166***	0.839***	1.100*	-0.125***	-0.087	-0.175***
	Income level 4	1.327***	0.963	1.273***	-0.142***	0.025	-0.161***
	Income level 5	0.990	1.008	0.987	0.054	0.078	0.024
Province	Province	Yes	Yes	Yes	Yes	Yes	Yes
Year	Year	Yes	Yes	Yes	Yes	Yes	Yes

Note: * p < 0.1, ** p < 0.05, *** p < 0.01

^a^ Annual healthcare costs are the natural logarithm of actual costs

### Heterogeneity analysis of parameter estimates for different age groups

For young adults (18–44), overweight/obesity had no additional healthcare costs compared to normal-weight individuals, and underweight individuals had higher utilisation of inpatient services (OR = 0.706, p < 0.05). For middle-aged adults (44–59), overweight/obese individuals had higher utilisation (OR = 1.107, p < 0.05) and costs (β = 0.133, p < 0.01) of non-inpatient services, as well as utilisation (OR = 1.148, p < 0.01) and costs (β = 0.145, p < 0.01) of healthcare services. Underweight was associated with a higher cost of non-inpatient services (β = 0.103, p < 0.1). For older adults (60 and above), overweight/obese individuals had higher utilisation of non-inpatient services (OR = 1.207, p < 0.01) and costs (β = 0.151, p < 0.01), and utilisation of healthcare services (OR = 1.169, p < 0.05) and costs (β = 0.130, p < 0.01). Underweight did not increase additional healthcare service utilisation and costs. (Table 5).

**Table 5:** Estimation of healthcare utilisation and expenditure on the heterogeneity of the age group

Type of characteristic	Annual healthcare utilisation [OR]			Annual healthcare costs^a^ [β]		
	Non-inpatient	Inpatient	Total healthcare	Non-inpatient	Inpatient	Total healthcare
Panel A: Overweight/obesity						
Age group						
18–44	0.937	1.016	0.929	-0.022	-0.034	-0.014
45–59	1.107**	1.098	1.148***	0.133***	0.012	0.145***
65 and above	1.207***	1.060	1.169**	0.151***	0.034	0.130***
Panel B: Underweight						
Age group						
18–44	1.119	0.706**	1.056	0.092	0.219	0.027
45–59	0.989	1.062	0.991	0.103*	-0.120	0.096
60 and above	1.015	1.045	0.982	-0.043	0.033	-0.014

Note: * p < 0.1, ** p < 0.05, *** p < 0.01

### Overweight/obesity attributable fraction (OAF) prediction

Based on the estimation results from the two-part model, we further predicted the healthcare costs caused by abnormal weight, including actual and counterfactual values, from which OAFs were calculated (Table 6). Using overweight/obesity as an illustration, the predicted value of healthcare cost for overweight/obesity groups is 149.429 USD, whereas the counterfactual value is 143.600 USD (assuming that the weight of obese or overweight people is normal). In other words, the impact of overweight/obesity on healthcare cost per capita is an increase of 5.829 USD, accounting for 3.90% of individual healthcare cost. The OAF of healthcare costs for overweight/obese individuals was 7.28% for middle-aged adults and 6.48% for older adults.

**Table 6:** Estimated annual healthcare costs attribute to overweight/obesity (China. 2012–2018)

Overweight/obesity	Predicted expenditure	Counter factual expenditure	Expected cost	OAF	p-value
**Panel A**					
Non-inpatient	97.726	93.518	4.208	4.31	< 0.001
Healthcare	149.429	143.600	5.829	3.90	< 0.001
**Panel B: Age group**					
**45–59**					
Non-inpatient	93.320	87.204	6.116	6.55	< 0.001
Healthcare	135.495	125.631	9.864	7.28	< 0.001
**60 and above**					
Non-inpatient	158.728	146.132	12.595	7.93	< 0.001
Healthcare	271.142	253.570	17.572	6.48	< 0.001

Note: All expenditures are based on 2018 CNY, adjusted for CPI from 2012 to 2018, and converted to USD based on an average exchange rate of 6.6174 in 2018; Only significant results were reported

## Discussion

Based on nationally representative datasets and econometric models, this study estimates the most recent values for the impact of abnormal weight on healthcare costs in Chinese adults. The results show that the prevalence of overweight and obesity among Chinese adults is comparable to that reported in the China Nutrition and Chronic Disease Report (2020), validating the representativeness of the data and the reliability of the findings. To the best of our knowledge, this is the most comprehensive and nationally representative study of overweight/obese and underweight people in China using the most recent data.

Healthcare costs for overweight/obese people have increased significantly in recent years. Using data from the China Health and Nutrition Survey (CHNS) from 2000 to 2009, a study found that the per capita medical cost of overweight and obesity was 6.18 CNY (0.90 USD), equivalent to 24.35 billion CNY (3.53 USD) of annual national medical expenditure [32], which is significantly lower than the estimate in this study. In addition, overweight/obese adults had significantly higher healthcare costs compared to adults of normal weight, with an OAF value of 3.90%. Notably, the utilisation rate and cost value of hospitalisation in the attributable costs of overweight/obesity were not significantly different from those with normal weight. Shi et al. (2011), using data from the China Health and Retirement Longitudinal Study (CHARLS), found that there were no significant differences in inpatient utilisation or inpatient costs for overweight/obese individuals over the age of 45 [32]. In accordance with the findings of this study, more additional costs for overweight/obese individuals are primarily non-inpatient costs.

Further, heterogeneity analyses by age groups revealed that middle-aged (45–59 years) and older adults (≥ 60 years) are more affected by overweight/obesity and their non-inpatient costs may be more related to outpatient and medication costs associated with chronic diseases [34–36]. Examples include cardiovascular disease treatment and prevention [37, 38] and anti-inflammatory medications [39, 40].

Lifestyle intervention is the first-line treatment for obesity in China, but research into its clinical efficacy is still largely insufficient [41]. Interventions for overweight and obesity must be regarded systematically and comprehensively for the time being. Firstly, improving public perception of unhealthy weight and the health risks associated with overweight and obesity is a prerequisite for promoting healthy lifestyles [42]. Secondly, the most effective intervention for obese individuals is the provision of readily accessible professional advice, including effective interventions developed by healthcare facilities, such as planned physical activity, healthy eating, and cognitive behavioral therapy [43]. Improving the abnormal weight status of Chinese population depends not only on self-perceptions of weight, risk perceptions, and a better grasp of existing interventions, but also on an awareness of the interdependence of biological, social, and political settings [41, 44]. For instance, Xiong et al. (2021) demonstrated that in recent decades, individuals tended to purchase low-nutrition, high-energy food and drink at a lower price. This transition in consumption had a substantial impact on the diet health of individuals, especially for those with low socioeconomic status [6]. Consequently, governments should allocate more financial resources towards food, with the aim of increasing the price of unhealthy foods and decrease the cost of healthy foods rich in fiber. Equally essential environmental drivers include urbanization, urban planning and built environments, food systems, and natural environments that shape obesity risk factors at the individual level [45, 46].

On the other hand, the results of underweight individuals did not show a statistically significant difference, which could be attributed to the small sample size of underweight individuals in the dataset. This suggests that more targeted studies on underweight people are warranted in future studies. Current relative economic inequality in the world impedes efforts to improve the nutrition of underweight individuals [47, 48]. Relative economic inequalities in societies undermine nutrition in numerous ways, including public education, diet, physical activity, food systems, health infrastructure, etc. [49, 50]. Additionally, relative economic inequality may explain why the distribution of underweight populations varied by location [51]. Low-income countries prioritize overweight, obesity and diet-related chronic diseases, and governments frequently lack adequate nutrition coordination [52, 53]. To estimate the spatial concentration of underweight people, a detailed study of the food and nutritional state of the population is required immediately. Accordingly, nutritional resources should be reallocated to the regions and populations most affected.

The study has some limitations. Firstly, this study focuses on individuals over the age of 18 and lacks a sample of minors. The self-reported height and weight of the respondents may differ marginally from the real situation. Secondly, obesity-related complications may occur in overweight/obese individuals, but due to the limited biological indicators, this study was unable to identify and answer the question of which characteristics of individuals are most likely to develop obesity-related complications, regardless of their weight status. This is what we can investigate and study in the future. Finally, due to the lack of information, indirect medical costs associated with abnormal weight were not considered in this study, which may lead to an underestimation of the economic burden.

## Conclusion

The study aimed to examine the impact of abnormal weight on the likelihood and cost of healthcare utilisation among Chinese adults. The results showed that among Chinese adults, overweight/obese individuals were more likely to utilise healthcare services than normal-weight individuals, especially non-inpatient services. In addition, overweight/obesity increases healthcare costs for middle-aged and older adults to varying degrees. These findings have substantial implications for how governments and healthcare institutions allocate resources for disease prevention and treatment.

## Data Availability

The data used in this paper is publicly available and could be accessible via the website of China Family Panel Studies (http://www.isss.pku.edu.cn/cfps/). The data used during the current study are available from the first author or corresponding author on reasonable request.
